# Accurate Detection of Carcinoma Cells by Use of a Cell Microarray Chip

**DOI:** 10.1371/journal.pone.0032370

**Published:** 2012-03-01

**Authors:** Shohei Yamamura, Shouki Yatsushiro, Yuka Yamaguchi, Kaori Abe, Yasuo Shinohara, Eiichi Tamiya, Yoshinobu Baba, Masatoshi Kataoka

**Affiliations:** 1 Health Research Institute, National Institute of Advanced Industrial Science and Technology (AIST), Takamatsu, Kagawa, Japan; 2 Division of Protein Expression, Institute for Genome Research, University of Tokushima, Tokushima, Japan; 3 Department of Applied Physics, Graduate School of Engineering, Osaka University, Suita, Osaka, Japan; 4 Department of Applied Chemistry, Graduate School of Engineering, Nagoya University, Furo-cho, Chikusa-ku, Nagoya, Japan; VTT Technical Research Centre of Finland and University of Turku, Finland

## Abstract

**Background:**

Accurate detection and analysis of circulating tumor cells plays an important role in the diagnosis and treatment of metastatic cancer treatment.

**Methods and Findings:**

A cell microarray chip was used to detect spiked carcinoma cells among leukocytes. The chip, with 20,944 microchambers (105 µm width and 50 µm depth), was made from polystyrene; and the formation of monolayers of leukocytes in the microchambers was observed. Cultured human T lymphoblastoid leukemia (CCRF-CEM) cells were used to examine the potential of the cell microarray chip for the detection of spiked carcinoma cells. A T lymphoblastoid leukemia suspension was dispersed on the chip surface, followed by 15 min standing to allow the leukocytes to settle down into the microchambers. Approximately 29 leukocytes were found in each microchamber when about 600,000 leukocytes in total were dispersed onto a cell microarray chip. Similarly, when leukocytes isolated from human whole blood were used, approximately 89 leukocytes entered each microchamber when about 1,800,000 leukocytes in total were placed onto the cell microarray chip. After washing the chip surface, PE-labeled anti-cytokeratin monoclonal antibody and APC-labeled anti-CD326 (EpCAM) monoclonal antibody solution were dispersed onto the chip surface and allowed to react for 15 min; and then a microarray scanner was employed to detect any fluorescence-positive cells within 20 min. In the experiments using spiked carcinoma cells (NCI-H1650, 0.01 to 0.0001%), accurate detection of carcinoma cells was achieved with PE-labeled anti-cytokeratin monoclonal antibody. Furthermore, verification of carcinoma cells in the microchambers was performed by double staining with the above monoclonal antibodies.

**Conclusion:**

The potential application of the cell microarray chip for the detection of CTCs was shown, thus demonstrating accurate detection by double staining for cytokeratin and EpCAM at the single carcinoma cell level.

## Introduction

Circulating tumor cells (CTCs) are known as the cells that have detached from a primary tumor and are circulating in the bloodstream, and the invasion of other tissues by them may occur very early during tumor development [1]. The presence of CTCs in the bloodstream supports the “seed and soil” theory of metastasis formation [2]. Although CTCs are as few as 1 cell per 10^9^ hematologic cells in the blood [3], these cells were shown to play an important role in the metastatic spread of cancer [4]. Thus the detection of CTCs would be expected to provide a powerful tool for cancer prognosis, diagnosis of minimal residual disease, assessment of tumor sensitivity to cancer drugs, and personalization of anticancer therapy [5]. Furthermore, highly sensitive and specific identification of CTCs could be useful for the early diagnosis of invasive cancers [2]. The CellSearch System™ (Veridex™, Raritan, NJ), which is based on immunomagnetic cell selection and enrichment by use of ferrofluid nanoparticles coated with anti-EpCAM (epithelial cell adhesion molecule, CD326) antibodies and the use of anti-CD45 antibody to discriminate leukocytes, is the only US Food and Drug Administration (FDA)-approved CTC diagnosis system on the market. The enriched population is stained with anti-cytokeratin antibody to discriminate between epithelial cells and contaminating leukocytes. Recently, a microfluidic platform capable of efficient and selective separation of CTCs from peripheral whole blood by using the interaction of CTCs with antibody-coated microposts was developed [3].

**Figure 1:**
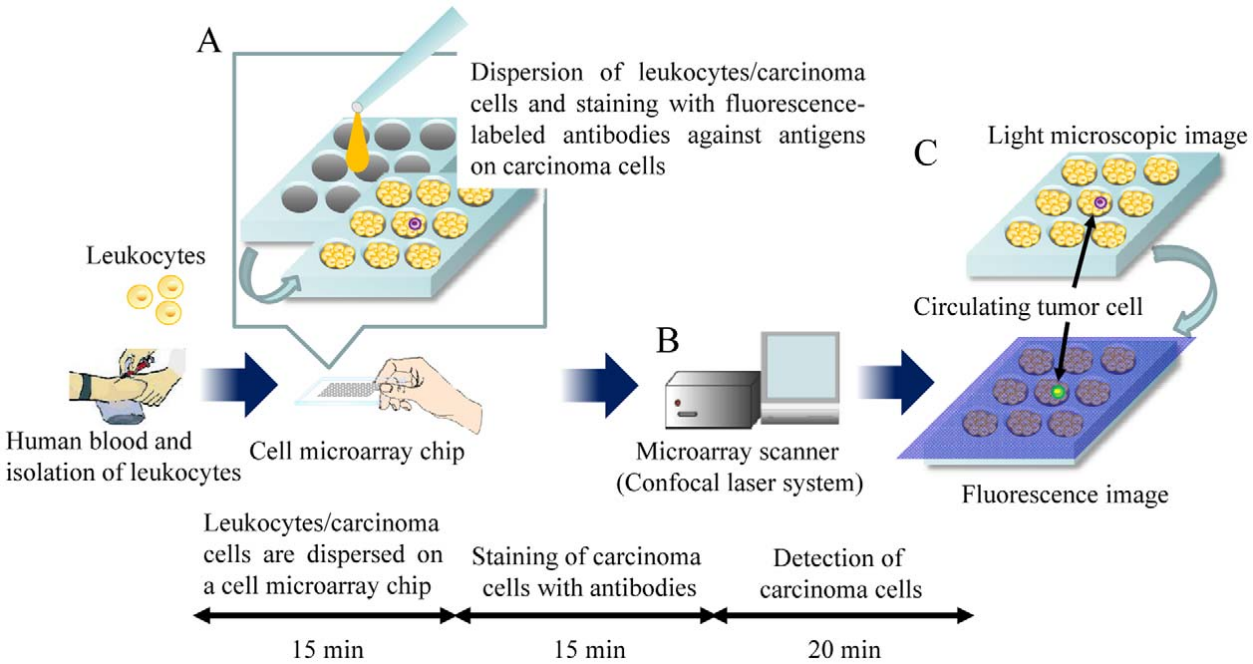
Schematic process for detection of CTCs on a cell microarray chip. (A) Human leukocytes/carcinoma cells are dispersed on a cell microarray chip, followed by 15 minutes' standing to allow the cells to settle down into the microchambers, and are then stained with fluorescence-labeled antibodies against carcinoma cells. (B) Fluorescence-positive CTCs are detected by using a microarray scanner with a confocal fluorescence laser. (C) The target CTCs are analyzed quantitatively at the single-cell level.

Microchip technologies have been expected to allow high-throughput and highly sensitive analysis of the function of individual cells [6]. In a previous study of ours, we developed a single-cell microarray chip for the analysis of antigen-specific single B-cells [7]. Furthermore, thereafter we developed a high-throughput screening and analysis system for the detection of malaria-infected erythrocytes, this system allowing high sensitivity and short time of operation and involving a cell microarray chip made from polystyrene with over 20,000 individually addressable microchambers [8]. Presently we employed this cell microarray chip system for the detection of human lung adenocarcinoma cells among leukocytes by staining with antibodies specific for epithelial cells or tumor cells. Our cell microarray chip was improved to allow the regular dispersion of hematologic cells and carcinoma cells to form a monolayer in the microchambers; and analysis of the cells following incubation with protein-staining fluorescent dyes was then carried out by use of a microarray scanner for the detection of the presence of fluorescence-positive carcinoma cells among the leukocytes (Fig. 1). In this study, we showed the potential of our cell microarray chip system for the accurate detection of carcinoma cells among leukocytes in a short time.

## Methods

### Construction of a cell microarray chip

**Figure 2:**
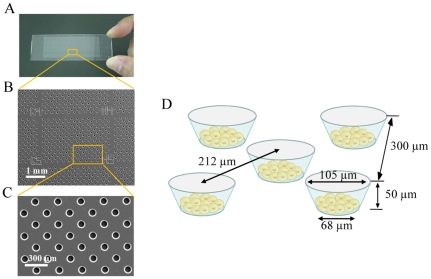
Construction of a cell microarray chip. (A) Photo of an actual cell microarray chip. (B, C) SEM images of a cell microarray chip. The cell microarray chip comprises 20,944 microchambers in a plastic slide on a glass slide. The cell microarray chip has 112 (14×8) clusters, each having 187 microchambers. (D) Each microchamber is 105 µm in upper diameter, 50 µm in depth, and has the shape of a frustum with a 68-µm diameter flat bottom for the accommodation of leukocytes as a monolayer.

As shown in Fig. 2, the cell microarray chip comprised 20,944 microchambers (105-µm upper diameter, 68-µm lower diameter, 50-µm depth, and spacing as indicated) and was made from polystyrene by the Lithographic Galvanoformung Abformung process by Starlight Co. Ltd. (Osaka, Japan) [8]. Each microchamber thus had the shape of a frustum. The polystyrene microarray chip was fabricated by injection molding with a nickel mold. The microarray chip contained 112 (14×8) clusters, each having 187 microchambers and a block number for easy confirmation of the presence of epithelial cells. The cell microarray chip surface was rendered hydrophilic by means of reactive ion-etching treatment by use of a SAMCO RIE system (SAMCO, Inc., Tokyo, Japan) to achieve cell confinement in the microchambers. The effect of reactive ion-etching exposure on the microarray chip surface was examined by measuring the contact angle of water on the chip surface by using a contact-angle meter (Kyowa Interface Science Co., Ltd., Saitama, Japan) [7], [8].

### Cell cultures and preparation for analysis on a cell microarray chip

Human bronchioalveolar carcinoma cells (NCI-H1650) were cultured in RPMI 1640 (Nakalai Tesque, Kyoto, Japan) containing 10% fetal bovine serum and antibiotics (100 U/ml penicillin-streptomycin ([GIBCO, Life Technologies Co., CA]) and 250 ng/ml Fungizone (GIBCO), and subsequently harvested by using trypsin. Human T lymphoblastoid leukemia cells (CCRF-CEM) were also cultured in a similar medium, and harvested by centrifugation. Cell suspensions of 7.5×10^4^, 7.5×10^3^, and 7.5×10^2^ NCI-H1650 cells/ml of medium were prepared, and 10 µl of each of these cell suspensions was added to 1.0 ml of 7.5×10^6^ CCRF-CEM cells/ml to give 0.01∼0.0001% NCI-H1650 samples, respectively. Five-hundred microliters of the respective cell samples were employed for the analysis on a cell microarray chip to detect the carcinoma cells.

For the preparation of carcinoma cell-spiked blood samples, 200 µl of 2.5×10^2^ cells/ml or 2.5×10^3^ NCI-H1650 cells were added to 10 ml of whole human blood (50 or 500 carcinoma cells spiked). For isolation of the carcinoma cells and leukocytes from the blood samples, 10 ml of the spiked whole blood was layered onto 10 ml of Polymorphprep™ (AXIS-SHIELD PoC AS, Oslo, Norway), and then centrifuged at 500×g, for 30 min at room temperature. By use of a Pasteur pipette, the leukocyte/carcinoma cell fraction was carefully harvested, washed with RPMI 1640 medium, and suspended in 2 ml of RPMI 1640 medium. The numbers of leukocytes were counted by use of a hemocytometer prior to the dispersion on a cell microarray chip. For the analysis on a cell microarray chip to detect carcinoma cells, 500-µl samples of the isolated leukocytes/carcinoma cells were employed as described below. For the double staining of cytokeratin and EpCAM, 200 µl of 12.5×10^4^ NCI-H1650 cells/ml were added to 1.0 ml of 7.5×10^6^ CCRF-CEM cells/ml or 10 ml of whole blood (2.5×10^4^ cells spiked) for the preparation of carcinoma cell-spiked blood samples.

### Detection of carcinoma cells on a cell microarray chip

For assessment of tight monolayers of the cultured cells in the microchambers, we examined leukocytes and carcinoma cells separately. First, 500 µl of 2.5×10^6^, 5.0×10^6^, 7.5×10^6^ or 1.0×10^7^ T lymphoblastoid leukemia/ml of RPM1 1640 medium was dispersed on a cell microarray chip, followed by 15 minutes' standing to allow the cells to settle down into the microchambers under gravitational force. For confirmation of tight monolayers of the carcinoma cells in the microchambers, 500 µl of 7.5×10^6^ carcinoma cells/ml of RPM1 1640 was dispersed on a microarray chip, followed by 15 minutes' standing. Then, excess cells on the chip surface were removed by gentle washing with RPM1 1640 medium, and the microchambers were examined by light microscopy.

For the analysis of carcinoma cell-spiked blood samples, 500 µl of leukocytes/carcinoma cells isolated with Polymorphprep™ were dispersed onto a cell microarray chip, and confinement of the cells as monolayers in the microchambers was performed as described above.

For the staining of cytokeratin in the dispersed cells in the microchambers, 500 µl of PE-labeled anti-cytokeratin monoclonal antibody (BD Biosciences, CA) solution (1∶ 50 dilution in 0.05% Triton X-100/PBS), which is specific for cytokeratins 7 and 8 (Ex: 543 nm, Em: 573 nm), was dispersed onto the cell microarray chip and allowed to react for 15 min; and then the chip surface were washed with RPMI 1640 medium. For the double staining of cytokeratin and EpCAM, the cell staining solution was prepared by adding 20 µl of PE-labeled anti-cytokeratin monoclonal antibody solution and 20 µl of APC-labeled anti-EpCAM monoclonal antibody (Biolegend, CA, Ex: 633 nm, Em: 660 nm) solution to 1 ml of 0.05% Triton X-100/PBS. Then 500 µl of this solution was dispersed onto the cell microarray chip and permitted to react for 15 min, and then the chip surface was washed with RPMI 1640 medium. For the staining of CD45, which is antigen present on all human leukocytes, 500 µl of APC-labeled anti-CD 45 monoclonal antibody (BD Bioscience, Ex: 633 nm, Em: 660 nm) solution (1∶ 200 dilution in 0.05% Triton X-100/PBS) was dispersed onto the cell microarray chip and allowed to react for 15 min; and then the chip surface was washed with RPMI 1640 medium. For the staining of cell membranes, the cells in each microchamber were incubated for 15 min with 500 µl of DiD (Invitrogen, Life Technologies Co., CA, Ex: 644 nm, Em: 665 nm) solution (1∶ 1000 dilution in 0.05% Triton X-100/PBS), and then the chip surface was washed with RPMI 1640 medium.

Each cell microarray chip was scanned for 20 min with a confocal laser-based fluorescence microarray scanner, CRBIO IIe (Hitachi Software Engineering Co., Ltd., Tokyo, Japan). This system exhibits a resolution of 10 µm and a sensitivity of <0.1 fluorescent molecule/µm^2^, and is fitted with filters with emission wavelengths of 532 and 635 nm. The fluorescence intensity of spots with a 15-µm diameter, nearly corresponding to individual carcinoma cells, was determined with DNASIS Array version 2.1 software (Hitachi Software Engineering Co., Ltd.), and the spots that exhibited fluorescence intensity that was 10 times above that of unstained cells were taken to be positive ones in antibody staining. For scatter-plot analysis of carcinoma cell-spiked blood samples, 12 spots with a 15-µm diameter in the each microchamber in 3 cluster areas representing 561 microchambers (total of 6732 spots) were examined.

### Statistical analysis

The number of fluorescence-positive cells was determined for each cell sample. Data were expressed as the mean ± standard error (SE) of at least 3 different experiments.

### Ethics

This study was approved by the Institutional Review Board of the National Institute of Advanced Industrial Science and Technology regarding the use of human derivatives for biomedical research, and by the Ethics Committee of University of Tokushima. All subjects provided written informed consent for the collection of samples and subsequent analysis.

## Results

### Dispersion of carcinoma cells and leukocytes on a cell microarray chip

**Figure 3:**
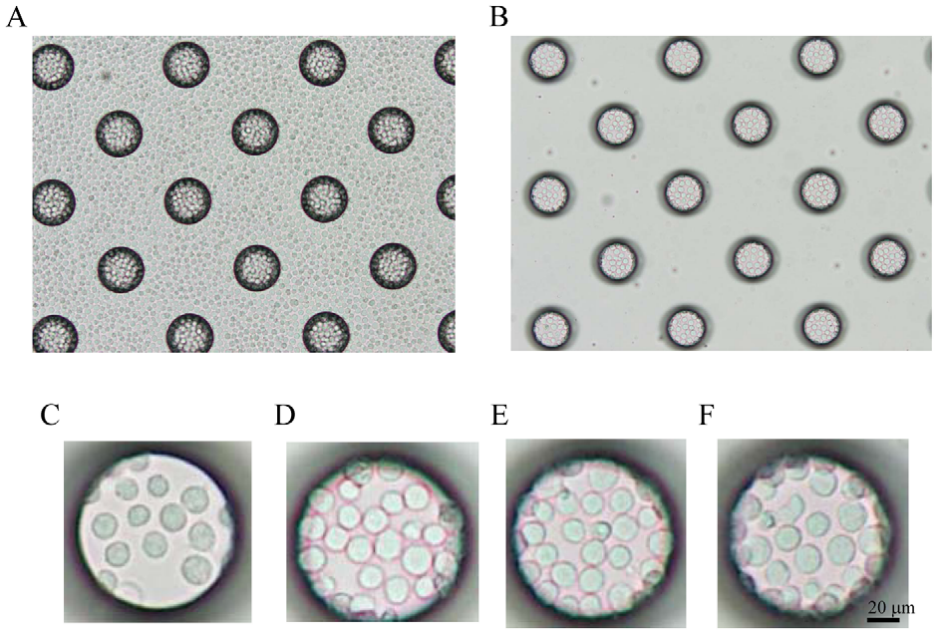
Dispersion of T lymphoblastoid leukemia cells on a cell microarray chip and confinement in the microchambers. (A, B) Photographic light microscopic images of T lymphoblastoid leukemia cells incubated on a cell microarray chip before (A) and after (B) washing of the chip surface. (C–F) Photos of microchamber appearance after washing when T lymphoblastoid leukemia suspensions of 2.5×10^6^ (C), 5.0×10^6^ (D), 7.5×10^6^(E) or 1.0×10^7^ (F) cells/ml were applied to the microarray chip. Concentrations of 7.5×10^6^ cells/ml of T lymphoblastoid leukemia and above afforded tight confinement and formation of a monolayer in the microchambers. (Bar: 20 µm).

**Figure 4:**
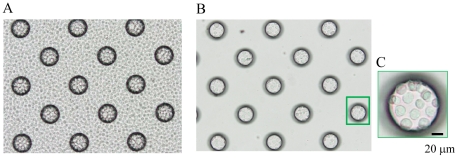
Dispersion of carcinoma cells on a cell microarray chip and confinement in the microchambers. (A, B) Photographic light microscopic images of carcinoma cells on a cell microarray chip before (A) and after (B) washing of the chip surface. (C) Carcinoma cells showed tight confinement and had formed a monolayer in the microchamber when a concentration of 7.5×10^6^ cells/ml was used. (Bar: 20 µm).

**Figure 5:**
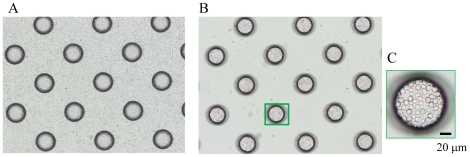
Dispersion of leukocytes isolated from whole blood on a cell microarray chip and confinement in the microchambers. (A, B) Photographic light microscopic images of leukocytes on a cell microarray chip before (A) and after (B) washing of the chip surface. (C) The leukocytes showed tight confinement and had formed a monolayer in the microchamber. (Bar: 20 µm).

To achieve the confinement of carcinoma cells and leukocytes in the microchambers, we optimized the hydrophilicity of the microarray chip surface by means of reactive ion-etching exposure [7], [8]. Eighty-second exposure gave appropriate hydrophilicity to the chip surface, and the cells could settle into each microchamber (data not shown). For the formation of a monolayer of cells on the bottom surface of the microchambers after washing, the cell microarray chip was designed to have microchambers with a 105-µm upper diameter, 50-µm depth, and a frustum shape (Fig. 2). The suspension of T lymphoblastoid leukemia was dropped onto the chip by using a pipette, and then the leukocytes settled down under gravitational force and adhered to the chip surface as multilayers (Fig. 3A). After the chip surface had been washed with medium by use of a pipette, only those leukocytes that had adhered to the bottom surface of each microchamber as a monolayer remained (Fig. 3B–F). We observed that the number of leukocytes confined to the microchambers depended on the concentration of leukocytes introduced onto the cell microarray chip, and leukocytes were tightly confined when a concentration of 7.5×10^6^ cells/ml of cell suspension or higher was used (Fig. 3E, F). The number of confined leukocytes was determined to be 29±2 (mean ± standard error (SE)) per microchamber (n = 40) with 7.5×10^6^ cells/ml of cell suspension (Fig. 3E). So, over 600,000 leukocytes were dispersed as a monolayer in the microchambers on a cell microarray chip when the cell suspension of 7.5×10^6^ cells/ml was used. A similar dispersion of bronchioalveolar carcinoma cells as a monolayer in the microchambers was observed with a cell suspension of 7.5×10^6^ cells/ml (Fig. 4). Similarly, for leukocytes isolated from whole blood, monolayer formation in the microchambers was confirmed (Fig. 5). The number of confined leukocytes was determined to be 89±4 (mean ± SE) per microchamber (n = 40); i.e., about 1,800,000 leukocytes were dispersed as a monolayer in the microchambers on a cell microarray chip.

### Fluorescence detection of T lymphoblastoid leukemia and carcinoma cells in the microchambers

**Figure 6:**
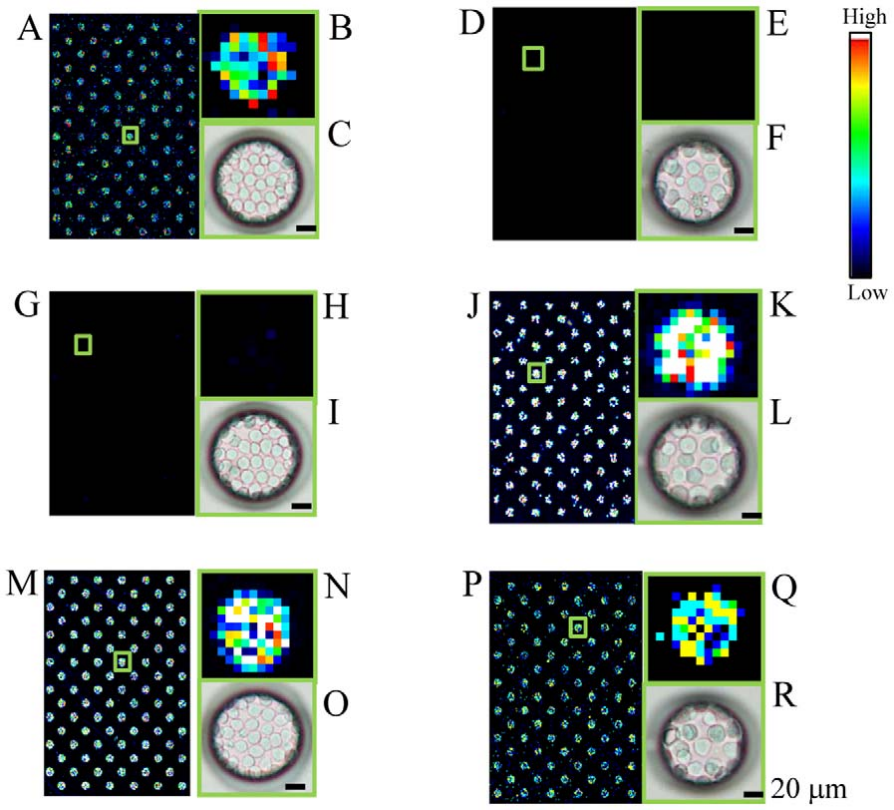
Fluorescence detection of T lymphoblastoid leukemia and carcinoma cells in the microchambers. (A,D) Scanning images of T lymphoblastoid leukemia (A) and carcinoma cells (D) stained with APC-labeled anti-CD 45 monoclonal antibodies in 128 microchambers. (B, E) Magnified views of the boxed regions in “A” and “D,” respectively. (C, F) Light microscopic images of microchambers in “B” and “E,” respectively, showing tight confinement of the cells as a monolayer. (G, J) Scanning images of leukocytes (G) and carcinoma cells (J) stained with PE-labeled anti-cytokeratin monoclonal antibodies in 128 microchambers. (H, K) Magnified views of the boxed regions in “G” and “J.” (I, L) Light microscopic images of microchambers in “H,” “K,” respectively, showing cells tightly confined as a monolayer. (M, P) Scanning images of leukocytes (M) and carcinoma cells (P) stained with DiD in 128 microchambers. (N, Q) Magnified views of the boxed regions in “M” and “P,” respectively. (O, R) Light microscopic views of microchambers in “N” and “Q,” respectively, showing tight confinement of cells as a monolayer. (Bar: 20 µm). Color scale at the right represents the intensity of fluorescent emission.

Scanning images of T lymphoblastoid leukemia stained with APC-labeled anti-CD 45 monoclonal antibody on a cell microarray chip are shown in Fig. 6A, B. All microchambers contained fluorescence-positive cells (Fig. 6A), and high-magnification images of the microchambers showed that fluorescence-positive pixels were tightly confined (Fig. 6B). Light microscopic observation also showed that the cells were tightly confined as a monolayer (Fig. 6C). Scanning images of carcinoma cells stained with APC-labeled anti-CD 45 monoclonal antibody on a cell microarray chip are shown in Fig. 6D, E. No fluorescence-positive cells were observed in the microchambers (Fig. 6D, E), but the light microscopic view revealed the presence of a monolayer of tightly confined carcinoma cells (Fig. 6F). By staining of leukocytes with PE-labeled anti-cytokeratin monoclonal antibody, no fluorescence-positive ones were observed in the microchambers (Fig. 6G, H), though tightly confined monolayers were seen by light microscopy (Fig. 6I). Fluorescence-positive carcinoma cells were observed by staining with the PE-labeled anti-cytokeratin monoclonal antibody in the microchambers (Fig. 6J, K), with tight monolayers confirmed by light microscopy (Fig. 6L). There was variation in the staining intensity of the carcinoma cells, and the fluorescence intensity of the light blue pixels (Fig. 6K) was over 10 times higher than that of those in the case of leukocytes (Fig. 6H). By staining with the anti-CD45 or anti-cytokeratin antibody and scanning with the microarray scanner, the fluorescence intensity of the stained cells was 10 times higher than that of the non-stained cells. These results indicate the validity of using the fluorescence- labeled anti-CD45 and anti-cytokeratin antibodies for the discrimination of the carcinoma cells among the leukocytes in the microchambers. By DiD staining, all microchambers were shown to be occupied by leukocytes (Fig. 6M, N) or carcinoma cells (Fig. 6P, Q, respectively), and tight monolayers were also confirmed by light microscopy (Fig. 6O, R, respectively).

### Detection of carcinoma cells in leukocyte cell suspensions on a cell microarray chip

**Figure 7:**
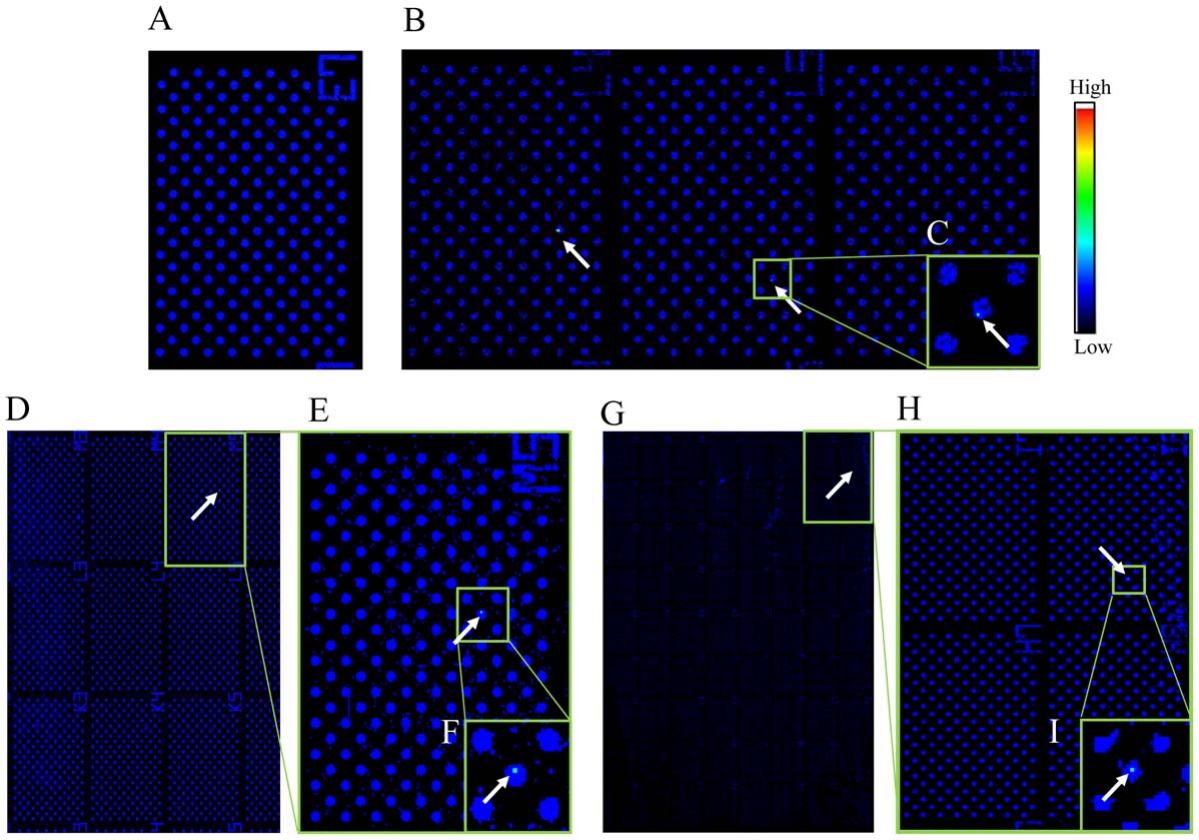
Detection of carcinoma cells among cultured T lymphoblastoid leukemia on a cell microarray chip. (A–I) Scanned images of leukocytes/carcinoma cells on a cell microarray chip obtained with the microarray scanner. (A) Negative control (no carcinoma cells). (B, D, G) Carcinoma cells (0.01, 0.001, and 0.0001%) were scanned in 3, 9, and 64 clusters, respectively, on the cell microarray chip. (C, E, F, H, I) Magnified views of the boxed regions. Color scale represents the intensity of fluorescent emission.

Fluorescent cytokeratin-positive carcinoma cells were observed among T lymphoblastoid leukemia in the microchambers with a cell mixture having 0.01%, 0.001%, and 0.0001% spiked carcinoma cells (Fig. 7B–I), the total numbers of fluorescence-positive carcinoma cells from independent experiments being 99.3±2.6, 9.7±0.3, and 1.0±0.0 (n = 3), respectively, in the whole microchamber area, as determined with DNASIS Array software. The percentage of carcinoma cells was determined from the following formula: [(number of fluorescence-positive cells/1,000,000 leukocytes)×100]. High-magnification images of microchambers containing fluorescence-positive carcinoma cells were obtained (Fig. 7C, F, I). The percentages of epithelial carcinoma cells in the mixture were calculated to be 0.00993±0.00026%, 0.00097±0.00003%, and 0.0001±0.0%, respectively. These calculated mixture levels were well consistent with the levels obtained practically. A good correlation between the calculated percentages and expectations was observed according to simple regression analysis (R^2^ = 1.0).

**Figure 8:**
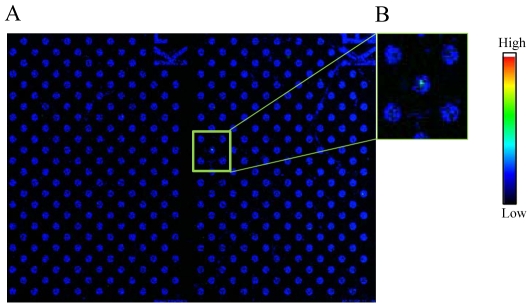
Detection of carcinoma cells among leukocytes/carcinoma cells isolated from whole blood and introduced onto a cell microarray chip. (A) Scanned images of cells on a cell microarray chip, obtained with the microarray scanner. The cells were immunostained with PE-labeled anti-cytokeratin monoclonal antibody. (B) Magnified view of the boxed region. Color scale represents the intensity of fluorescent emission.

Fluorescence-positive cells among the leukocytes isolated from whole blood that had been spiked with the cancer cells were detected with PE-labeled anti-cytokeratin monoclonal antibody (Fig. 8). The rate for spiked carcinoma cells in whole blood was determined by using the following formula: [(50 cells or 500 cells/calculated leukocytes)×100] (Table 1). Each spike rate estimated with the cell microarray chip was determined from the following formula: [(number of fluorescence-positive carcinoma cells/total number of leukocytes confined in the whole microchamber area)×100]. The estimated spike rates were well consistent with the ones obtained practically. A good correlation between the 2 results was observed according to simple linear regression analysis (R^2^ = 0.9994).

**Table 1:** Analysis of spiked carcinoma cells in human whole blood on a cell microarray chip.

Sample*	number of Leukocytes	spike rate	number of cells**	number of leukocytes	number of carcinoma cells	estimated spiked rate
	(/10 ml)	(%)	(/microchamber)	(/chip)	(/chip)	(%)
1	25,600,000	0.000195	89±3.1	1,860,000	4	0.000215
2	26,200,000	0.000191	90±2.7	1,880,000	4	0.000213
3	16,800,000	0.000298	87±2.2	1,820,000	5	0.000275
4	27,800,000	0.000180	89±4.2	1,860,000	3	0.000161
5	25,400,000	0.00197	89±5.2	1,860,000	38	0.00204
6	26,200,000	0.00191	87±2.3	1,820,000	37	0.00203
7	27,600,000	0.00181	89±3.7	1,860,000	34	0.00183

*Samples no. 1 to 4 were spiked with 50 carcinoma cells; and 5 to 7, were spiked with 500 carcinoma cells,

**Ten microchambers were randomly selected on a cell microarray chip, and the number of carcinoma cells in each of the chambers was counted.

### Double staining of carcinoma cells on a cell microarray chip

**Figure 9:**
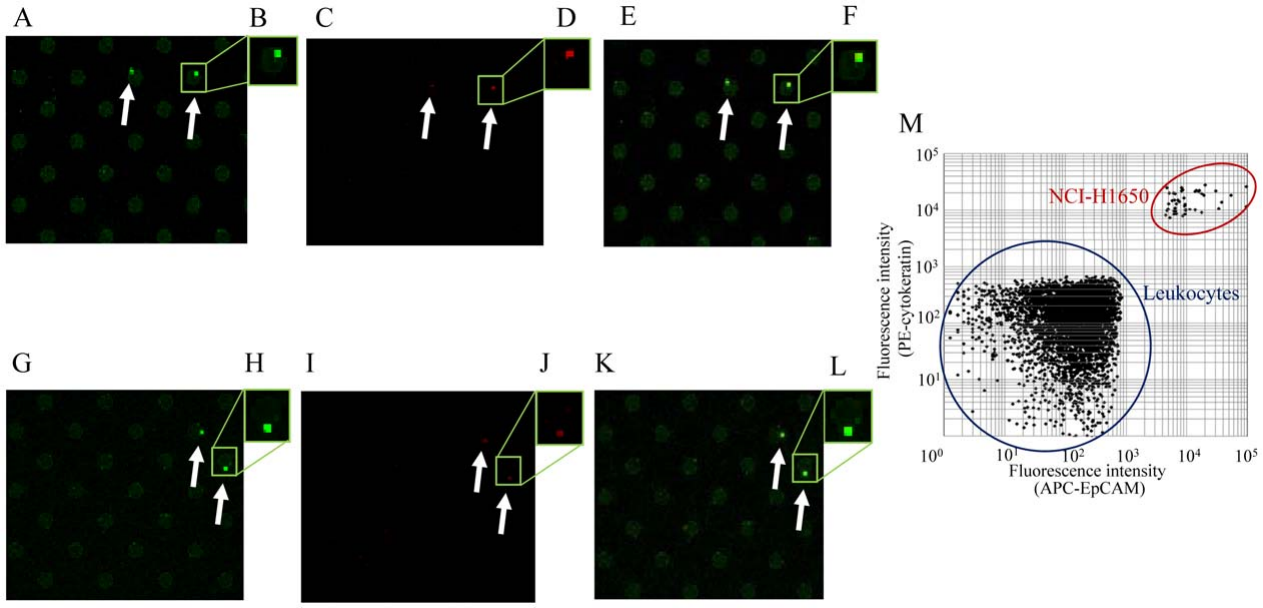
Scanned images of double-stained cells. (A–L) Cultured T lymphoblastoid leukemia (A–F) and leukocytes isolated from whole blood (F–L) were stained with PE-labeled anti-cytokeratin monoclonal antibody (A, G) and APC-labeled anti-EpCAM monoclonal antibody (C, I). (E, K) Merged images identify doubly-positive carcinoma cells in each panel. Magnified views of the boxed regions (B, D, F, H, J, L). Scatter-plot analysis of 3 cluster areas representing 561 microchambers in a cell microarray chip (Fig. 9M).

For verification of carcinoma cells in the microchambers, double staining with 2 monoclonal antibodies specific for carcinoma cells was performed (Fig. 9). Fluorescent images of carcinoma cells among cultured T lymphoblastoid leukemia cells stained with PE-labeled anti-cytokeratin monoclonal antibody (Fig. 9A) or APC-labeled anti-EpCAM monoclonal antibody (Fig. 9C) were obtained, and the merged image identified the doubly positive carcinoma cells (Fig. 9E). Likewise, the same results were obtained for the whole blood spiked with carcinoma cells (Fig. 9G, I, K, respectively). Scatter plots demonstrated that the cytokeratin-positive cells were also EpCAM positive (Fig. 9M). Several times higher fluorescence intensity was observed than that of leukocytes with either staining. These results indicate the usefulness of the cell microarray chip and staining with 2 antibodies specific for verification of carcinoma cells among cultured T lymphoblastoid leukemia and/or leukocytes isolated from whole blood.

## Discussion

For the sensitive detection of CTCs in peripheral blood, an enrichment process is frequently employed, e.g., filtration, density gradient or immunomagnetic [2]. In the filtration and density gradient methods, there are the disadvantages of low sensitivity and/or low specificity. The CellSearch™ system, which is an FDA-approved CTC diagnosis system on the market, employs an automated and immunomagnetic method for quantification of CTCs. It uses ferrofluid nanoparticles coated with anti-EpCAM antibodies to isolate and enrich for CTCs that express the EpCAM molecule on their cell surface; and anti-cytokeratin antibodies are used to identify CTCs. So, the simultaneous expression of EpCAM and cytokeratin on the same CTCs is the key factor for accurate detection of CTCs. Several prospective studies using patients with metastatic breast, colorectal or prostate cancer have shown that above a cut-off value of 5 CTCs per 7.5 ml of blood have poor survival prospects [9]; and changes in CTC counts during systemic therapy can serve as an early marker for a positive or negative response to treatment [10]. Variation in the molecular characteristics of CTCs is well known [9]. For example, there are 5 subtypes of human breast cancers based on global gene expression profiling, i.e., normal-like, basal, HER2-positive, and luminal A and B [11]. Normal-like cells lack EpCAM expression, and these cells are thus missed when CTCs are captured by an EpCAM-based method such as the CellSearch System™ [10], [11].

The unique microfluidic platform named “CTC-chip” consisting of an array of 78,000 microspots coated with anti-EpCAM antibodies was reported to isolate CTCs [3]. EpCAM-positive cells are captured on the microspots when whole blood is passed through the microfluidic device, and these cells are detected by analysis of the expression of tumor markers [2], [3]. Capture efficiency is dependent on the level of EpCAM expression, and the limitation is that the CTC-chip is suitable only for EpCAM-positive cells [2], [12]. The most common epithelial markers are cytokeratins, which are cytoskeletal proteins expressed in epithelial cells; and EpCAM, a cell-adhesion molecule is present on epithelial cells as well [2]. Cytokeratins are specific markers of epithelial cells and can be used as one criterion for carcinoma detection [13]. As was shown in Figs. 7 and 8, the use of fluorescence-labeled anti-cytokeratin antibody for the detection of carcinoma cells among cultured T lymphoblastoid leukemia or leukocytes isolated from whole blood was validated. Highly accurate detection of CTCs was obtained because only fluorescence-labeled anti-cytokeratin antibody-binding cells among mono-layered leukocytes were targeted and detected with the confocal fluorescence laser scanning system. The importance of analyzing the expression of cell-surface antigens and/or gene expression for the distinct prognostic and therapeutic characteristics for cancer is well known [12], [14]–[16]. As was shown in Fig. 9, this cell microarray technology is quite suitable for the analysis of protein expression on cell membranes by staining with multi-antibodies for the characterization of carcinoma cells; and the staining procedure is easy because the fluorescence-labeled antibody solution is simply dispersed onto the cell microarray chip and the chip washed prior to scanning. Previously we reported the recovery of single cells by using a micromanipulator and a cell microarray chip having microchambers with a diameter of 10 µm, with gene analysis performed by PCR for the cell characterization [7]. Although we did not recover the carcinoma cells from the microchambers in the present study, for gene analysis by PCR it would be easy to recover them by using a micromanipulator; because the present cell microarray chip has microchambers with a diameter of 105 µm.

Interestingly, 3 times more leukocytes could be accommodated in the microchambers on the cell microarray chip with cells isolated from whole blood (Fig. 5) than with cultured T lymphoblastoid leukemia, CCRF-CEM (Fig. 3). This observation must have been due to the smaller cell diameter of the former. CTCs are very rare, with an expected concentration as low as 1 cell per 10^5^–10^7^ mononuclear cells [2]. The CellSearch System can detect 1 CTC in 7.5 ml of whole blood [17], meaning that a maximum of 1×10^8^ leukocytes can be analyzed with this system. As just 1.8×10^6^ leukocytes isolated from whole blood can be analyzed on a single cell microarray chip, over 50 chips would be needed to analyze statistically the numbers of leukocytes. Obviously, this would not be realistic in clinical use. So, we need to increase the number of microchambers on a cell microarray chip and/or improve the design of the microchambers to accommodate a larger number of leukocytes. Although our present cell microarray chip technology is insufficient in ability for analyzing large cell numbers, we have shown the potential of the cell microarray chip for accurate detection of CTCs by determining protein expression on cancer cell membranes by multi-antibody staining.
